# The Role of PSMA PET Imaging in the Classification of the Risk of Prostate Cancer Patients: A Systematic Review on the Insights to Guide an Active Surveillance Approach

**DOI:** 10.3390/cancers16061122

**Published:** 2024-03-11

**Authors:** Francesco Dondi, Alessandro Antonelli, Nazareno Suardi, Giorgio Treglia, Francesco Bertagna

**Affiliations:** 1 Nuclear Medicine, ASST Spedali Civili di Brescia and Università degli Studi di Brescia, 25123 Brescia, Italy; francesco.bertagna@unibs.it; 2 Department of Urology, Azienda Ospedaliera Universitaria Integrata Verona, University of Verona, 37134 Verona, Italy; alessandro.antonelli@aovr.veneto.it; 3 Department of Urology, Spedali Civili di Brescia, 25123 Brescia, Italy; nazareno.suardi@unibs.it; 4 Nuclear Medicine, Imaging Institute of Southern Switzerland, Ente Ospedaliero Cantonale, 6500 Bellinzona, Switzerland; giorgio.treglia@eoc.ch; 5 Department of Nuclear Medicine and Molecular Imaging, Lausanne University Hospital, University of Lausanne, 1011 Lausanne, Switzerland; 6 Faculty of Biomedical Sciences, Università della Svizzera Italiana, 6900 Lugano, Switzerland

**Keywords:** PSMA, prostate-specific membrane antigen, positron emission tomography, PET, prostate cancer, active surveillance, nuclear medicine

## Abstract

**Simple Summary:**

The prognosis of prostate cancer (PCa) patients and their best therapeutic approach are related to the risk-based classification of this neoplasm since subjects with higher risk could have a higher incidence of recurrence. In addition, patients with low- and intermediate-risk PCa could benefit from active surveillance (AS). Prostate-specific membrane antigen (PSMA) positron emission tomography (PET) imaging has demonstrated its value for the assessment, prognostic role, and ability to guide the therapy of PCa. The aim of this systematic review was to assess the role of PSMA PET in guiding the correct classification of low-to-intermediate risk PCa subjects and the AS approach. Insights on the value of this imaging modality in these settings have emerged; however, further research in this field is necessary to clearly define the role of PSMA PET.

**Abstract:**

Background: active surveillance (AS) is a suitable strategy for patients with prostate cancer (PCa). Prostate-specific membrane antigen (PSMA) positron emission tomography (PET) imaging is an established tool used to assess PCa. The aim of this review was to evaluate the role of PSMA imaging to guide correct risk-based classification and the AS approach in PCa patients. Methods: The Scopus, Embase, Web of Science, Cochrane Library, and PubMed/MEDLINE databases were screened to find relevant published articles. Results: 1774 articles were revealed with the literature search. A total of 1764 articles were excluded after applying exclusion criteria (data not within the field of interest, preclinical papers, conference proceedings, reviews, or editorials). Ten studies were finally included in the review, revealing that PSMA PET could have the ability to guide risk-based classification of PCa and the choice of AS, and to guide the execution of biopsies for the research of high-grade PCa, therefore precluding AS. Conclusion: this systematic review underlined a possible role of PSMA PET imaging in patients with PCa by correctly re-classifying them on the basis of their risk and guiding AS.

## 1. Introduction

Prostate cancer (PCa) is one of the most frequent forms of neoplasm affecting men. Its incidence is related to the age of the patients, with the highest peak between 65 and 74 years and with an estimated 1,600,000 cases and 366,000 deaths annually [1,2]. To obtain prognostic and survival information and to choose the best therapeutic option, patients affected by PCa are stratified into low-, intermediate-, and high-risk groups based on different clinicopathological characteristics such as the Gleason score (GS) derived from biopsy results, serum prostate-specific antigen (PSA) levels, and clinical stage [3,4]. As mentioned above, this classification has an extremely important impact on the prognosis of these subjects, since patients classified in higher-risk groups tend to have a higher incidence of local or systemic recurrence despite optimal therapy [5]. Imaging plays an important role in the diagnostic assessment of PCa as well as in the definition of the risk category of each patient. In this setting, many different imaging modalities can be used to evaluate these subjects, starting from multiparametric magnetic resonance imaging (mpMRI) which has been recognized as the most precise imaging method for PCa detection and for T stage assignment [6,7]. More recently, prostate-specific membrane antigen (PSMA) positron emission tomography (PET) imaging performed with both PET/MRI or PET/CT tomographs has emerged as a promising and useful modality for both staging and restaging settings of PCa patients, as well as for the assessment of therapeutic response and therefore of prognosis prediction [8,9,10,11]. In particular, it has been underlined that PSMA PET/CT is a promising tool in the management of low- to intermediate-risk PCa, improving risk stratification and identifying patients at risk of pathological upstaging [12].

The therapeutic approach to PCa can be influenced by many different factors and several treatment modalities are currently available. In particular, not all PCa patients need prompt treatment since several randomized clinical trials underlined that the risk of death from other causes can supersede the risk of death from PCa [3,13,14]. In this setting, one of the therapeutic approaches that can be offered to subjects with low- or intermediate-risk neoplasms is represented by active surveillance (AS) [15]. AS is defined as a structured program that employs monitoring and delayed intervention in the management of low-risk PCa and this approach currently represents the first choice for the majority of patients with low-risk or favorable intermediate-risk diseases [16]. Different factors such as health conditions, disease characteristics, side effects, life expectancy, and patient preferences are taken into account when choosing AS [17]. Additionally, AS provides different advantages over radical treatments such as preservation of erectile function, decreased costs of treatment, avoidance of over-treatment of no life-threatening disease, with preservation of quality of life and normal activities. On the other hand, AS may be affected by significant flaws, such as the misclassification of patients with more advanced diseases with the possibility of missing the therapeutic window. Moreover, AS can translate into increased anxiety in patients and frequent medical checks [18]. Therefore, the selection of patients suitable for AS may be improved by the latest technological advances, especially in terms of imaging. Therefore, the aim of this systematic review was to evaluate the role of PSMA PET imaging in guiding the correct risk-based classification and AS approach in patients affected by PCa.

## 2. Materials and Methods

The present review was performed by following the PRISMA guidelines but was not registered in any public registry.

### 2.1. Search Strategy

A wide literature search of the PubMed/MEDLINE, Scopus, Web of Science, Cochrane Library, and Embase databases was performed in order to identify published articles addressing the role of PSMA PET/CT in the clinical setting of AS in PCa patients. The algorithm used for the research was: “psma AND (‘pet’/exp OR pet) AND (‘follow up’/exp OR ‘follow up’ OR (follow AND up) OR ‘surveillance’/exp OR surveillance OR ‘monitoring’/exp OR monitoring)”.

The search had no beginning date limit and was updated until 15 December 2023. Articles considered for the review were written in the English language. In addition, conference proceedings, editorials, preclinical studies, or reviews were not included in the present review. Moreover, to find additional papers to expand our search, the references of the retrieved articles were screened.

### 2.2. Study Selection

The titles and abstracts of the retrieved articles were independently reviewed by two researchers (G.T. and F.D.), who also reviewed the full-text version of the identified articles to determine their eligibility for inclusion.

### 2.3. Quality Assessment

Risk of bias and applicability concern assessments were carried out using Quality Assessment of Diagnostic Accuracy Studies version 2 (QUADAS-2) evaluation [19]. Quality assessments were performed independently by two reviewers.

### 2.4. Data Extraction

Two reviewers independently evaluated the retrieved studies to collect relevant information. For each study included in the review, data concerning the basic information of the study such as first author name, year of publication, country of origin, design of the study, radiotracer used, and number of patients were collected. Furthermore, information about the type of PET tomograph used, the mean activity of the injected tracer, the type of imaging analysis used, and the main results were also collected. The main findings of the articles included in this review are reported in the Results section.

## 3. Results

### 3.1. Literature Search

**Figure 1:**
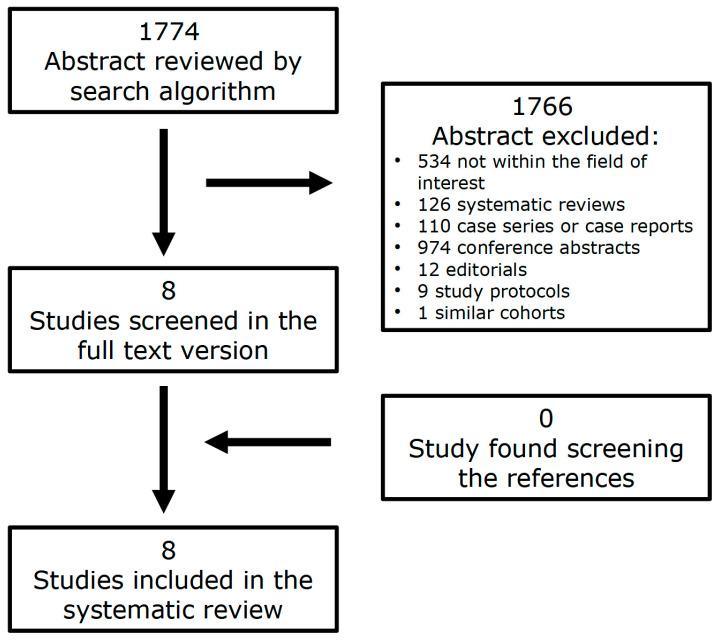
Flowchart of the research of eligible studies on the role of PSMA PET/CT in the clinical setting of AS in PCa patients.

The literature search from the databases retrieved a total of 1774 articles. After reviewing the titles and abstracts, 1766 of them were excluded for different reasons: 534 because the reported data were not within the field of interest of this review, 126 were systematic reviews, 110 were care series or case reports, 974 were conference abstracts, 12 were editorials, nine were study protocols, and one study had the same cohort as another paper. Consequently, eight studies addressing the role of PSMA PET/CT in the clinical setting of AS were selected and the full texts were retrieved [20,21,22,23,24,25,26,27]. No additional studies were found after analyzing the reference lists of these articles (Figure 1).

**Figure 2:**
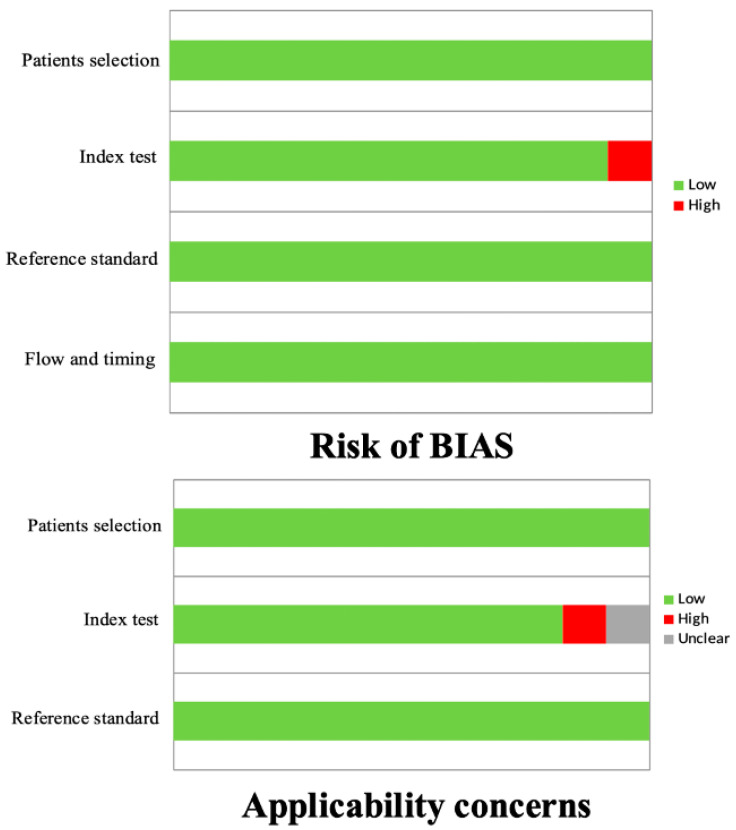
QUADAS-2 quality assessment for risk of bias and applicability concerns for the studies considered in the review.

In general, the quality assessment using QUADAS-2 evaluation underlined the presence of a low risk of bias in most of the domains for all the studies included in the review (Figure 2).

Among the total number of studies included in the systematic review, three were retrospective [22,23,26], whereas five had a prospective design [20,21,24,25,27]. In terms of radiopharmaceuticals used, five studies were performed using [^68^Ga]Ga-PSMA-11 [20,21,23,24,27], a single study with [^18^F]F-PSMA-1007 [22], a study with both [^68^Ga]Ga-PSMA-11 and [^18^F]F-DCFPyL [26], while in one study, the tracer used was not specified and referred to as a general [^68^Ga]Ga-PSMA [25], although different papers published by the same group used [^68^Ga]Ga-PSMA-11. Moreover, six studies were performed using a PET/CT tomograph [21,23,24,25,26,27] and two studies used PET/MRI [20,22]. The main characteristics of the studies and their results are briefly presented in Table 1 and Table 2.

### 3.2. Role of PSMA PET Imaging in Guiding the Risk Classification of PCa and AS

The first study to analyze the possible role of [^68^Ga]Ga-PSMA-11 PET/MRI in the clinical setting of risk classification of PCa subjects and AS was published by Grubmüller et al. [20], who included 122 PCa patients and evaluated the staging performances of this imaging modality and its impact on therapeutic decisions. PET/MRI demonstrated high accuracy in both T and N staging (82.3% and 93.0%, respectively), with an impact on clinical treatment decisions in a high number of patients. Particularly, 19 (15.6%) subjects with minimal or no detectable tumor burden at PET/MRI and with favorable intermediate- or low-risk features were treated with AS. Interestingly, all of them had no primary treatment at the last available follow-up. Similarly, a paper by Sasikumar et al. [21] investigated the role of [^68^Ga]Ga-PSMA-11 PET/CT for the assessment of patients harboring neoplasms in a cohort of 118 subjects with suspected PCa, revealing that it could act as a gate-keeper for the selection of patients who should undergo immediate deeper clinical investigations or could be kept on AS. In particular, 51 (43%) subjects had a negative PET/CT scan and could, therefore, avoid being biopsied, being only followed up or having had a negative biopsy. In contrast, most of the patients with a positive scan had a diagnosis of PCa.

[^18^F]F-PSMA-1007 PET/MRI was used by Liu et al. [22] to evaluate 62 patients with either suspected or defined PCa. Overall, imaging was able to change decisions in 26 (41.9%) subjects, including 11/28 (39.3%) in the group with suspected PCa with a decrease in the AS approach (from 20 to 9) and an increase in prostate biopsies (from 8 to 19). During follow-up, for 14/28 (50%) patients, the AS approach was confirmed and, interestingly, all 11 subjects who had a negative PET/MRI were put on AS and no PCa was found until the last available follow-up.

Raveenthiran et al. [23] performed an interesting study that aimed to evaluate the ability of both mpMRI and [^68^Ga]Ga-PSMA-11 PET/CT to predict tumor localization and the presence of a Gleason score (GS) ≥ 3 + 4 on robot-assisted laparoscopic radical prostatectomy (RALP); the second aim of this paper was to evaluate whether these imaging modalities could predict subjects with a GS < 3 + 4 at biopsy who may be upgraded to ≥3 + 4 on RALP. The authors included a large sample of 1123 men and revealed that index lesion and multifocal tumor detection rates were similar between mpMRI and PET/CT. Furthermore, combining both, an index GS ≥ 3 + 4 was identified in 92% of the patients with a significantly high accuracy. A significant relationship between standardized uptake value (SUVmax) and GS was reported (*p* < 0.01); moreover, only 10% of GS ≤ 3 + 4 patients with an SUVmax < 5 were upgraded to ≥4 + 3, compared to 90% if the SUVmax was >11, which was itself the value with the best area under the 183 curve (AUC). Interestingly, mpMRI and SUVmax were reported to be significantly associated with an upgrade of GS to >4 + 3 on final RALP. As a whole, these insights permitted the authors to suggest that for patients suitable for AS with a SUVmax > 11 on PSMA PET/CT, a targeted biopsy of the tracer-avid area would be recommended to exclude high-grade Pca before endorsing follow-up.

Xue et al. [24] assessed the ability of [^68^Ga]Ga-PSMA-11 PET/CT and SUVmax to improve risk stratification in patients with intermediate-risk PCa. Two hundred and twenty men were studied, reporting that at biopsy, the median SUVmax of regions with >50% Gleason pattern 4 (GP4) was higher than regions with <50% GP4 (*p* < 0.001). These data were also confirmed with cut-off values of 20% and 10% for GP4 (*p* < 0.001 for both analyses). Multivariate analysis confirmed SUVmax as an independent predictor of the presence of GP4 at per-patient analysis for all three thresholds, with AUC > 0.70 for all of them. Moreover, segments with a pathological upgrading from biopsy to surgery had a significantly higher SUVmax compared to non-upgraded segments (*p* < 0.001) and SUVmax was a significant discriminator for upgrading with an AUC of 0.73. Additionally, patients with an unfavorable pathology after surgery had a higher SUVmax compared to those without such a characteristic (*p* < 0.001) and this parameter was again confirmed as a significant predictor (*p* < 0.001). All these data suggested that PET/CT findings can predict high GS and possibly add another layer of safety when stratifying patients for their eligibility for AS or treatment in favorable intermediate-risk subjects.

Pepe et al. [27] included 40 low-risk subjects to evaluate the diagnostic accuracy of [^68^Ga]Ga-PSMA-11 PET/CT for the assessment of clinically significant PCa (csPCa) (GG ≥ 2) in men enrolled in the AS protocol. Patients underwent both this imaging modality and mpMRI, revealing 9/40 (22.5%) and 18/40 (45%) lesions suspicious for Pca, respectively, which were then subjected to transperineal saturation prostate biopsies (SPBx). In 3/40 (7.5%) men, a clinically significant Pca (csPCa) was found and PSMA-targeted biopsies (TPBx) vs. mpMRI-TPBx vs. SPBx diagnosed 2/3 (66.6%) vs. 2/3 (66.6%) vs. 3/3 (100%) csPCa, respectively. In addition, mpMRI and [^68^Ga]Ga-PSMA-11 PET/TC demonstrated 16/40 (40%) vs. 7/40 (17.5%) false positive and 1 (33.3%) vs. 1 (33.3%) false negative results. Moreover, these imaging modalities revealed a diagnostic accuracy for the diagnosis of csPCa of 70.2% and 83.3%, respectively. More recently, Heetman et al. [25] enrolled 141 patients with the purpose of evaluating the risk stratification and selection of patients for AS with the addition of PSMA PET/CT. At a median assessment with PET/CT of 2 months after the start of AS, 45 (32%) patients had an additional biopsy due to the presence of a new tracer-avid lesion or due to an elevated SUVmax. In two (1.4%) patients, downgrading was observed, while in 13 (9%) subjects, upgrading was detected at the International Society of Urologic Pathology (ISUP) grade: nine grade group (GG) 2, two GG 3, one GG 4, and one GG 5. The number needed to scan (NNS) to detect one patient with upgrading was 11. Out of all the participants, PSMA PET/CT and targeted biopsies yielded upgrading most frequently in patients with negative MRIs. Out of patients who received additional PSMA-targeted biopsies, upgrading was most frequently found in those with higher PSMA densities and negative MRIs.

Jain et al. [26] assessed the role of PSMA PET/CT in AS in men newly diagnosed with low or favorable intermediate-risk PCa, including 30 patients submitted to both [^68^Ga]Ga-PSMA-11 and [^18^F]F-DCFPyL. In the 15 subjects with GG 1 disease at biopsy, seven were assigned to AS and all of them had mildly expressing lesions upon PSMA imaging. Moreover, five patients had RALP and three had a repeated biopsy, four of whom upgraded based on final prostatectomy specimens. Seven had concerning features at PET/CT such as the presence of MRI occult lesions, marked PSMA uptake, and extraprostatic extensions. Fifteen men had a GG 2 result at initial diagnostic biopsy and after performing PSMA PET/CT, four of them were submitted to AS while 11 underwent RALP. Eight men had the aforementioned concerning features at PET/CT. In the case of patients with abnormal PSMA findings, the corresponding prostatectomy revealed adverse pathological features; in the case of a patient who upgraded to GG 3, the corresponding PSMA imaging revealed marked uptake with an SUVmax of 8.42. Overall, 11/30 (37%) men were assigned to AS, and at follow-up, 10 of them remained in this group while 19/30 (63%) had definitive treatment. Moreover, 15/19 of these subjects had concerning PET/CT features and for nine of them, adverse pathological features were confirmed upon final prostatectomy. The key finding of the study was the demonstration that management was diverted to intervention for a significant proportion of men suitable for AS based on the combination of MRI and PSMA PET findings.

## 4. Discussion

PCa is one of the most frequent forms of neoplasm affecting men, with a variety of possible therapeutic approaches that could be offered to the patients depending on initial risk stratification and subsequent clinical follow-up. Nevertheless, the treatment strategy should be patient-centered and based on the specific features of each subject. As a matter of fact, not all PCa needs prompt treatment since, as previously mentioned, the risk of death from other causes can supersede the risk of death from non-clinically significant forms [3]. AS is one of the therapeutic approaches that could be offered to patients with favorable forms of PCa, in particular low- or intermediate-risk neoplasms or to those with specific clinical conditions [15]. This approach is a structured program that employs monitoring of the neoplasm, and imaging has a central role in this scenario. In particular, this is a clear and established modality with an important value for the follow-up of PCa subjects and its ability to possibly influence the management strategy of these patients has been underlined. In this setting, PSMA PET imaging has a prominent role in assessing PCa at both staging and restaging but also in the assessment of therapeutic response [8,9,28]. Therefore, its value for the guidance of AS has been studied in different papers.

The clinical process to assess PCa starts with its correct diagnosis. Obviously, a suspected neoplasm should not be treated or managed as diagnosed PCa. Interestingly, PSMA PET imaging has demonstrated a possible value in the setting of AS in subjects with suspected PCa. In particular, PET/MRI was able to change the clinical treatment decision of these patients, with a reduction in men sent to AS and an increase in biopsies. In particular, the importance of having a negative PET/MRI scan has been highlighted, given its ability to exclude the presence of PCa even after follow-up, therefore aiming for the correct selection of AS as a management strategy [22]. Subjects suspected to have PCa were also studied with PSMA PET/CT, emphasizing its ability to act as a gatekeeper to better define if a patient should undergo a deeper clinical assessment or could be sent to AS. Again, a negative scan excluded the presence of disease and could therefore be used as an indication for AS planning, while in most of the patients, a positive scan was related to the presence of a neoplasm [21].

The histopathologic grading of PCa according to GS and GG are major determinants that define the risk class of these patients, influencing their therapeutic pathway and approach [29]. In this scenario, a lack of consensus on the criteria for the selection of intermediate-risk patients suitable for AS is present since this group consists of highly heterogeneous patients. In addition, the careful selection of intermediate-risk PCa is fundamental as it is known that this neoplastic entity has worse outcomes compared to low-risk PCa on AS [12]. Insights on the role of PSMA PET imaging for the correct re-classification of PCa have emerged, aiding the correct framing of patients from low- and intermediate-risk to high-risk neoplasm, a change that can influence the prognosis of these subjects. In this setting, it has been reported that in patients on AS, PSMA PET/CT has the ability to modify the treatment strategy: the presence of areas of higher tracer uptake should be further studied with biopsy to exclude the presence of high-grade PCa before endorsing follow-up [23]. In addition, the value of the intensity of uptake has been underlined, revealing a correlation between SUVmax and high GS. Moreover, a high SUVmax (>11) was underlined as predictive of an upgrade of GS after RALP [23]. Similarly, evidence on the fact that the same semiquantitative PET/CT parameter is significantly elevated in patients with higher group patterns has been reported, with SUVmax thought to be an independent predictor of the presence of higher group patterns. Moreover, it has been reported that subjects and areas with higher SUVmax had pathological upgrading from biopsy to surgery and that this parameter was associated with an unfavorable pathology result [24]. Consequently, these findings suggest that PSMA PET/CT is somehow able to predict GS and therefore help in the risk stratification of patients for their eligibility for AS or treatment.

The comparison of PSMA imaging with mpMRI in the clinical settings of risk reclassification and AS has been performed and studies in some papers. The addition of PSMA PET/CT in the work-up of PCa during AS can lead to the performance of an additional biopsy due to the appearance of new tracer-avid lesions or due to an elevation of SUVmax values, resulting in an upgrade of the neoplasm risk class. Interestingly, PSMA PET/CT and targeted biopsies yielded upgrading most frequently in patients with negative MRI and, in patients with additional PSMA-targeted biopsies, upgrading was most frequently found in those with a higher PSMA density and a negative MRI [25]. Additionally, it has been reported that PSMA PET/CT was able to change the management approach of patients suitable for AS with low or favorable intermediate-risk PCa, resulting in the diversion of management to intervention based on some concerning imaging features such as the presence of MRI occult lesions, marked PSMA uptake, and extraprostatic extension [26]. Nevertheless, the combination of these two imaging modalities into PSMA PET/MRI revealed high diagnostic accuracy in the staging of PCa and the ability to change clinical treatment decisions in a significant number of patients, including a switch to the AS approach [20].

Despite the previous findings reported and underlined in this review, less enthusiastic results were demonstrated by other authors. In particular, in low-risk patients in AS protocols, a comparison between PSMA PET/CT and mpMRI revealed that the first imaging modality did not improve the detection of csPCa. Despite that, it demonstrated a lower rate of false positive findings compared to mpMRI [27].

Based on the findings collected in this review, a possible role for PSMA PET imaging for the evaluation of PCa patients has emerged, which can be used to guide the choice of AS. In this scenario, different and heterogeneous findings about the cost-effectiveness of performing PET/CT for the initial staging of PCa subjects have been reported. In some cases, increased costs were reported, while in others, cost savings were highlighted [30,31]. Interestingly, it has been stated that the positive effect of performing a PSMA PET/CT at staging was caused by abandoning unnecessary treatment in metastatic patients, while the negative effect was caused by a lower quality of life and high costs in the group of patients receiving false palliative treatment instead of potentially curative therapy [30]. In addition, costs for an accurate diagnosis using PSMA PET/CT seem to be reasonably low compared to the potential consequential costs of an inaccurate diagnosis [31]. As a consequence, the idea of including PSMA imaging in patients suitable for AS seems reasonable; however, even if the use of this imaging modality is appropriate from a health-economic perspective, prospective evaluation of patients at initial diagnosis needs to be performed in order to verify this fact [31].

Even though our findings revealed a possible role for PSMA PET imaging in the setting of AS, it is worth emphasizing that no specific prognostic data were evaluated and reported by the papers included in the review. As a consequence, new efforts and research are required to clearly assess this fact. In particular, the impact of subsequent metastasis-free survival or cancer-specific survival should be analyzed.

Some limitations, derived from the characteristics of the studies included, affect our systematic review. Firstly, some of the studies are characterized by a small cohort. Moreover, even if some papers had an acceptable number of patients, only a limited number of them were included in AS protocols. Lastly, four studies had a retrospective design. Based on these facts, no meta-analysis of the data retrieved could be performed.

## 5. Conclusions

In conclusion, this review underlined the possible role of PSMA PET imaging in patients with PCa on AS. In particular, this imaging modality could have the ability to correctly re-classify subjects on the basis of their risk, guide the choice of AS, and guide the execution of biopsies for the research of high-grade PCa, therefore precluding AS. However, further research in this field is necessary to clearly define the role of PSMA PET.

## Figures and Tables

**Table 1:** Characteristics of the studies considered for the review.

First Author	Ref. N.	Year	Country	Study Design	Radiopharmaceuticals	N. Pts.
Grubmüller B	[20]	2018	Austria, USA	Prospective	[^68^Ga]Ga-PSMA-11	122
Sasikumar A	[21]	2018	India	Prospective	[^68^Ga]Ga-PSMA-11	118
Liu A	[22]	2021	China	Retrospective	[^18^F]F-PSMA-1007	62
Raveenthiran S	[23]	2022	Australia, Sweden, New Zealand	Retrospective	[^68^Ga]Ga-PSMA-11	1123
Xue A	[24]	2022	Australia	Prospective	[^68^Ga]Ga-PSMA-11	220
Heetman J	[25]	2023	The Netherlands	Prospective	ns	141
Jain A	[26]	2023	Australia	Retrospective	[^68^Ga]Ga-PSMA-11, [^18^F]F-DCFPyL	30
Pepe P	[27]	2023	Italy	Prospective	[^68^Ga]Ga-PSMA-11	40

N.: number; Pts: patients; Ref.: reference; ns: not specified ([^68^Ga]Ga-PSMA); PSMA: prostate-specific membrane antigen.

**Table 2:** Results and main findings of the studies considered for the review.

First Author	Device	Mean Activity ± SD or Median (Range) (MBq)	PET Analysis	Main Findings
Grubmüller B	PET/MRI	ns	Qualitative	PSMA PET/MRI had high diagnostic performances in PCa staging, correctly identifying 97.5% of cancers with an accuracy of 82.5%, 85%, 79%, 94%, and 93% for T, T2, T3a, T3b, and N1 staging, respectively. It also provided important information for the development of a personalized therapeutic strategy with changes in overall treatment strategy in 28.7% of the subjects. In particular, 15.6% of the patients were submitted to AS.
Sasikumar A	PET/CT	100 ± 19	Qualitative and semiquantitative	PSMA PET/CT could select between suspected PCa patients who should undergo further investigation and those who could be kept on AS. In particular, 43% of the patients had a negative scan and could therefore avoid biopsy and be kept in follow-up or had a negative biopsy. Most of the subjects with a positive scan had a positive biopsy.
Liu A	PET/MRI	263 (164–353)	Qualitative	PSMA PET/MRI was a valuable tool with which to define PCa and tailor the management of the patients. In particular, management changes were reported in 39.3% of the subjects with suspected PCa with an increase in biopsy and a reduction in AS. All subjects with a negative PET/MRI were put on AS and no PCa was reported until the last follow-up.
Raveenthiran S	PET/CT	200	Qualitative and semiquantitative	Adding PSMA PET/CT to MRI could improve the detection of significant PCa, improving the ability to identify patients suitable for AS. Similar detection rates between the two imaging modalities (82% and 80%, respectively) were reported and, by combining them, a higher accuracy was underlined (92% for GS ≥ 3 + 4). Significant correlation between SUVmax and GS was demonstrated; in addition, for patients suitable for AS with an SUVmax > 11 on PSMA PET/CT, a targeted biopsy of the tracer-avid area should be recommended to exclude high-grade PCa.
Xue A	PET/CT	2/Kg ± 5% and 2/Kg ± 10%	Qualitative and semiquantitative	SUVmax was associated with GP4 and, therefore, PSMA PET/CT could improve risk stratification of favorable intermediate-risk PCa patients. Higher SUVmax for regions with >50% GP4 compared to regions with <50% was reported; the same findings were confirmed using 20% and 10% as reference thresholds. SUVmax was an independent predictor for the presence of GP4, a significant discriminator for upgrading after surgery, and a significant predictor of unfavorable pathology after surgery.
Heetman J	PET/CT	1.5/Kg	ns	PSMA PET/CT could improve PCa risk stratification and allow a better selection of AS patients. In particular, 32% of the subjects had an additional biopsy due to PET/CT findings and in 9% of the subjects, upgrading was detected. PET/CT and biopsies yielded upgrading frequently in MRI-negative patients.
Jain A	PET/CT	ns	Qualitative and semiquantitative	PSMA PET/CT could influence the management of patients with a new diagnosis of low or favorable intermediate-risk PCa before their consideration for AS. The presence of concerning features at PET/CT imaging or high SUVmax values were correlated to an upgrade of GG or to the presence of adverse pathological features after radical prostatectomy. Based on PET/CT findings, 37% of the men were submitted to AS.
Pepe P	PET/CT	144 ± 12	Qualitative and semiquantitative	PSMA PET/CT did not improve the detection rate of clinically significant PCa on biopsy; however, it demonstrated better diagnostic accuracy compared to mpMRI. In particular, mpMRI and PSMA PET/CT revealed that 45% and 22.5% of lesions were suspicious for PCa, respectively. PSMA-target biopsy diagnosed 66.6% of Pca compared to 66.6% and 100% of mpMRI-guided biopsy and transperineal saturation biopsy, respectively. mpMRI demonstrated 40% and 33.33% of FP and of FN results, respectively, while PET/CT revealed 17.5% of FP and 33.3% of FN findings, respectively.

PSMA: prostate-specific membrane antigen; ns: not specified; PET: positron emission computed tomography; CT: computed tomography; MRI: magnetic resonance imaging; AS: active surveillance; MBq: Megabecquerel; Kg: kilogram, PCa: prostate cancer; SD: standard deviation; GS: Gleason score, SUVmax: standardized uptake value; GP: Gleason pattern; GG: grade group; mpMRI: multiparametric MRI; FP: false positive; FN: false negative; ns: not specified.

## Data Availability

Data supporting the reported results can be found using the public PubMed/MEDLINE, Scopus, Web of Science, Cochrane Library, and Embase databases.
